# Multivariable Analysis of the Association Between Lumbar and Lumbosacral MRI-Diagnosed Spinal Pathologies and Pain in Dogs

**DOI:** 10.3390/ani15050761

**Published:** 2025-03-06

**Authors:** Roger Medina-Serra, Patricia López-Abradelo, Eliseo Belda, Holly Riding-Medina, Francisco G. Laredo, Rachel Marwood, Verónica Mortera, José I. Redondo

**Affiliations:** 1 Anaesthesia and Pain Management, North Downs Specialist Referrals, Bletchingley RH1 4QP, UK; patricia.lopez@ndsr.co.uk; 2 Escuela Internacional de Doctorado de la Universidad de Murcia, Programa en Ciencias Veterinarias, Universidad de Murcia, 30100 Murcia, Spain; 3 Departamento de Medicina y Cirugía Animal, Facultad de Veterinaria, Universidad de Murcia, 30100 Murcia, Spain; laredo@um.es; 4 Hospital Veterinario Universidad de Murcia, 30100 Murcia, Spain; 5 Diagnostic Imaging, North Downs Specialist Referrals, Bletchingley RH1 4QP, UK; holly.rider@ndsr.co.uk; 6 Diagnostic Imaging, CityU Veterinary Medical Centre, City University of Hong Kong, Kowloon, Hong Kong, China; 7 Neurology and Neurosurgery, North Downs Specialist Referrals, Bletchingley RH1 4QP, UK; veronica.mortera@ndsr.co.uk; 8 Departamento de Medicina y Cirugía Animal, Facultad de Veterinaria, Universidad Cardenal Herrera-CEU, 46115 Valencia, Spain; nacho@uchceu.es

**Keywords:** lumbar pain, lumbosacral pain, MRI, canine degenerative lumbosacral stenosis, spinal pathology, intervertebral disc disease, radiculopathy, pain management, dog

## Abstract

Lumbar and lumbosacral pain in dogs often involves multiple concurrent spinal pathologies, complicating the identification of primary pain generators. This study assessed the associations between MRI-diagnosed spinal pathologies and pain to provide clinically relevant insights for their diagnosis and management. MRI scans and clinical records of 518 client-owned dogs were retrospectively reviewed, documenting demographic data, pain status, and MRI findings. Multivariable logistic regression models evaluated the associations between spinal pathologies and pain, adjusting for age and weight. The intervertebral disc (IVD) extrusion was the primary pathology associated with lumbar pain, while radiculopathy had the strongest association with lumbosacral pain. Additional lumbosacral pathologies, including foraminal stenosis, IVD bulging, and IVD protrusion, were also significantly associated with pain. However, some dogs with MRI-diagnosed abnormalities showed no pain, whereas others with pain had no detectable MRI pathology, underscoring the need to interpret imaging within the clinical context. Pain responses observed during physical examination often overlapped between spinal and hip conditions, complicating diagnostic accuracy. These findings emphasise the importance of integrating imaging, clinical assessment, and targeted diagnostic techniques to improve pain localisation and treatment decisions, providing veterinarians with valuable data to refine the management of lumbar and lumbosacral pain in dogs.

## Simple Summary

Lumbar and lumbosacral pain in dogs is often linked to multiple concurrent spinal pathologies, making it difficult to identify the primary source of pain. This study investigated the associations between MRI-diagnosed spinal pathologies and pain in dogs to better understand which conditions are most relevant for clinical decision-making. By analysing MRI scans and clinical records from a large sample of dogs, we identified specific spinal conditions that were more frequently associated with pain, including intervertebral disc extrusion and radiculopathy. Our findings suggested that imaging alone is not always sufficient to determine pain sources, as some dogs with spinal abnormalities did not exhibit pain, while others with pain showed no clear MRI pathology. Additionally, we found that pain responses to clinical examination can overlap, making it difficult to determine whether the discomfort arises from the spine or the nearby joints, such as the hips. These findings highlight the need for a comprehensive approach that integrates imaging, clinical examination, and additional techniques to refine diagnosis and treatment strategies. This research provides veterinarians with valuable insights to improve pain management in dogs, ultimately enhancing their well-being and quality of life.

## 1. Introduction

Low back pain (LBP) is a term used in human medicine to describe the pain affecting the lumbar and lumbosacral regions, which on occasions radiates to the gluteal region and pelvic limbs [1]. This condition is multifactorial, involving a complex interplay of nociceptive, neuropathic, and nociplastic pain, as well as the central nervous system’s processing and modulation of pain, which is heavily influenced by behavioural, cognitive, and emotional factors. Pathologies such as intervertebral disc (IVD) disease, spinal stenosis, radiculopathies, facet joint degeneration, sacroiliac degeneration, and pathologies affecting the myofascial system are commonly associated with LBP. Furthermore, genetic predisposition, lifestyle, psychosocial factors, and trauma are significant risk factors for its development [1].

Magnetic Resonance Imaging (MRI) is widely regarded as the gold standard in imaging modality for assessing the majority of spinal pathologies due to its ability to visualise soft tissue structures, including the spinal cord, nerve roots, and intervertebral discs, and its non-invasive nature [2]. MRI plays a crucial role in identifying and characterising anatomical abnormalities associated with patient pain (pain generators), thereby enabling the development of targeted treatments tailored to specific pathologies [3]. However, these findings must be interpreted in the context of the clinical presentation, as MRI identifies pathologies, but does not necessarily correlate to pain generators, given that many patients with spinal abnormalities are asymptomatic [4,5,6,7].

Spinal pathologies, such as IVD degeneration and lumbosacral stenosis, are more commonly observed in ageing dogs and larger breeds [8,9,10,11,12,13]. In chondrodystrophic breeds, IVD degeneration predominantly affects the cervical and thoracolumbar regions, whereas in non-chondrodystrophic breeds, it more frequently involves the caudal cervical and lumbosacral regions, with occasional involvement of the thoracolumbar spine [11].

Caudal lumbar pain in dogs is commonly attributed to the broad diagnosis of degenerative lumbosacral stenosis (DLSS), characterised by a range of degenerative changes at the lumbosacral junction, commonly including intervertebral disc disease, hypertrophy of the interarcuate ligament, and facet joint disease. Collectively, these changes contribute to the compression of neural structures, with the cauda equina being compressed within the lumbosacral vertebral canal and the lumbosacral spinal nerves experiencing compression as they exit through the intervertebral foramina [8,10].

A key clinical challenge in the early assessment of caudal lumbar and lumbosacral pain in dogs is differentiating between the pain originating from the lumbosacral spine and pain associated with hip pathology. Pain elicited during hip extension is a common finding in both conditions, making it difficult to determine whether the discomfort stems from the hip joint or results from mechanical stress on the lumbosacral region [8,14,15]. This overlap is particularly relevant in the initial stages of diagnosis, where physical examination findings guide decision-making before advanced imaging is considered.

In this study, we investigated the relationships between MRI findings, demographic factors, and the presence or absence of lumbar and/or lumbosacral pain in a population of client-owned dogs presenting to a single referral veterinary hospital. Five hypotheses were proposed: (1) Lumbar/lumbosacral MRI spinal pathologies will be present in both patients with and without pain; however, their prevalence is expected to be significantly higher in patients presenting with pain; (2) older and heavier patients will be more likely to exhibit lumbar/lumbosacral MRI-pathology and pain; (3) specific lumbar/lumbosacral pathologies, including intervertebral disc (IVD) extrusions, foraminal stenosis, facet joint pathologies, and radiculopathies, will be significantly associated with lumbar and/or lumbosacral pain; (4) the pain elicited during hip extension will be associated with lumbosacral pain and pathology; and (5) the pain on lumbosacral palpation will not be associated with hip pain and pathology.

The objectives of this study were: (1) to evaluate the prevalence of lumbar/lumbosacral MRI pathologies in dogs with and without lumbar/lumbosacral pain and determine whether their prevalence is significantly higher in dogs presenting with pain; (2) to assess the associations between demographic factors, specifically age and weight, and the presence of lumbar/lumbosacral MRI pathologies and pain; (3) to investigate the relationship between specific MRI pathologies, such as IVD extrusions, foraminal stenosis, facet joint pathologies, and radiculopathies, and the presence of lumbar and/or lumbosacral pain; (4) to determine whether the pain elicited during hip extension is associated with lumbosacral pain and lumbosacral pathology; and (5) to examine whether the pain elicited during lumbosacral palpation is associated with hip pathology and hip pain.

## 2. Materials and Methods

### 2.1. MRI Selection

Magnetic Resonance Imaging (MRI) studies, acquired using a Canon 1.5 Tesla whole body unit at North Downs Specialist Referrals, were retrieved retrospectively (H.R.-M.) from the hospital database (PACS; Picture Archiving and Communication System) using the OsiriX (Pixmeo, Switzerland) and Horos software (Horos medical imaging software, version 3.3.4). Radiological studies were filtered by modality (MRI), species (dog), and description (spine, lumbar, and lumbosacral) before being reviewed. The retrospective search included the period between September 2017 and January 2025. Only studies containing water-excitation sequences encompassing the caudal lumbar and the cranial sacral region, as well as sagittal and transverse T1W (T1-weighted) and T2W (T2-weighted) sequences, were selected. A Microsoft Excel spreadsheet was created to document the date of the MRI acquisition, along with the associated patient’s hospital number, breed, sex, age, and weight at the time of acquisition.

### 2.2. Clinical History

After the initial selection, the respective clinical histories and reports provided by the attending veterinary specialists at the time of the MRI were retrieved and reviewed (P.L.-A.; R.M.-S.). Only the patients with MRI reports authored by a board-certified veterinary radiologist (ECVDI) or an ECVDI resident under the supervision of a board-certified radiologist were included in the study.

### 2.3. Variables Recorded

In addition to the patient’s hospital number, breed, sex, age, and weight at the time of acquisition, several dichotomous (YES/NO) variables were recorded. These include the presence of lumbar/lumbosacral pain (YES/NO) as described by the referring veterinarian at the time of the MRI (L/LS_Pain_RVS), the presence of lumbar/lumbosacral pain (YES/NO) as described by the attending referral veterinary specialist at the time of the MRI (L/LS_Pain_Ref), and an overall pain status variable indicating lumbar/lumbosacral pain (L/LS_Pain), recorded as YES if either or both of the previous two variables were marked as YES. Pain elicited during clinical examination at the referral centre, conducted at the time of the MRI, was recorded. This included pain elicited during lumbar (Pain_Lumbar) and lumbosacral (Pain_Lumbosacral) palpation, as well as during tail dorsiflexion (Pain_Tail) and hip extension (Hip_Extension).

Dichotomous variables were also recorded for MRI-diagnosed pathologies. These included the presence of lumbar pathology (MRI_Lumbar), lumbosacral pathology (MRI_Lumbosacral), and an overall MRI pathology status (MRI_Pathology), recorded as YES if either or both of the previous two variables were marked as YES. Additionally, specific pathologies were documented, including cauda equina compression (Cauda_Equina), intervertebral disc disease (IVD_disease), intervertebral disc (IVD) extrusion (IVD_Extrusion), IVD protrusion (IVD_Protrusion), IVD bulging (IVD_Bulging), foraminal stenosis (Foraminal_Stenosis), radiculopathy (Radiculopathy), facet joint pathology (Facet_Joint_Pathology), sacroiliac degeneration (Sacroiliac_Pathology), hip pathology (Hip_Pathology), spinal tumour (Spinal_Tumour), spinal nerve tumour (Spinal_Nerve_Tumour), and spinal subluxation (Spinal_Subluxation). Dogs were classified as having no hip pathology if their MRI field of view included the coxofemoral joints and no abnormalities were noted by the reporting radiologist. When separate radiographic or computed tomography examinations specifically evaluated the hip joints, those results were used to confirm or exclude hip pathology. Patients lacking any imaging or records clarifying the status of the hip joints were not included in the analyses incorporating hip pathology. Additional pathologies were documented, and for the patients without spinal pain observed during the examination, the final diagnosis or reasons for undergoing the MRI were also recorded.

### 2.4. Statistical Analysis

All statistical analyses were performed (R.M.-S.; J.I.R.) using the R programming language (R Studio, version 2024.12.0 + 467). The approach involved descriptive statistics, regression modelling, and additional sub-analyses to test the study hypotheses.

#### 2.4.1. Descriptive Study

Initially, a descriptive study was performed to summarise demographic, imaging and clinical characteristics of the study population.

#### 2.4.2. Agreement Between Referring and Referral Veterinary Surgeons on Overall Pain Status

Cohen’s kappa coefficient was calculated to evaluate the agreement between the referring and referral veterinary surgeons regarding the overall pain status.

#### 2.4.3. Association Between Overall MRI Pathology and Overall Pain Status

A chi-squared test of independence incorporating a Cramér’s V was performed to evaluate the association between the overall MRI pathology and overall pain status.

#### 2.4.4. Age and Weight on Overall MRI Pathology and Overall Pain Status

Age and weight were categorised into distinct groups for analysis. Age was classified into four categories: adolescence (0 to 24 months), mature adult (25 to 84 months), senior (85 to 144 months), and geriatric (145 months and above). Weight was grouped into three categories: small (0 to 10 kg), medium (11 to 25 kg), and large (26 kg and above). Logistic regression models were employed to evaluate the association between these categorical predictors, overall MRI pathology, and overall pain status.

#### 2.4.5. Specific MRI Pathologies and Pain Status

Binary logistic regression models were used to evaluate the associations between specific MRI-diagnosed pathologies and pain status. The analyses were stratified as follows:

- Specific MRI pathologies and overall pain status: associations between each MRI-diagnosed pathology and the presence of overall pain.
- Specific lumbar MRI pathologies and lumbar pain: associations between the pathologies identified in the lumbar region and the presence of lumbar pain, excluding all patients with a lumbosacral pathology.
- Specific lumbosacral MRI pathologies and lumbosacral pain: associations between the pathologies identified in the lumbosacral region and the presence of lumbosacral pain, excluding all patients with lumbar pathology.

#### 2.4.6. Clinical Examination Findings and Pain and Pathology

Two binary logistic regression models were developed to evaluate the associations between the specific clinical pain examination findings and pain or pathology outcomes:

- Pain during hip extension and lumbosacral pain/pathology: the associations between pain during hip extension and the combined presence of lumbosacral pain and pathology, excluding cases of hip pathology.
- Pain during lumbosacral palpation and hip pain/pathology: associations between pain during lumbosacral palpation and the combined presence of hip pain and pathology, excluding cases of lumbosacral pathology.

For all analyses, the categories with an insufficient number of cases were excluded. All analyses were conducted after removing the cases with missing values. Variables with a *p*-value of less than 0.05 were considered statistically significant. For logistic regression models, variables with high collinearity (>5) were excluded. Model performance was assessed using discriminative ability (C-statistics), calibration (Hosmer–Lemeshow test), and model fit (Akaike Information Criterion [AIC] and Deviance). Results were reported as the number of cases (n) and percentages (%), median (range), odds ratio (OR), 95% confidence interval (CI), and *p*-value (*p*), as appropriate.

## 3. Results

### 3.1. MRI Selection and Clinical Records

From the initial 1405 MRI studies retrieved and reviewed, 561 studies containing water-excitation sequences encompassing the caudal lumbar and cranial sacral regions, as well as sagittal and transverse T1W (T1-weighted) and T2W (T2-weighted) sequences, were selected. The associated patient data, including hospital number, breed, sex, age, and weight at the time of MRI acquisition, were recorded in a spreadsheet. Following the initial selection, 29 cases were excluded due to the absence of a radiologist’s report, and 14 cases were excluded due to missing referral report from the referring veterinarian at the time of the MRI. As a consequence, a total of 518 patients met the inclusion criteria for the study and were included in the statistical analysis.

### 3.2. Descriptive Study

**Figure 1:**
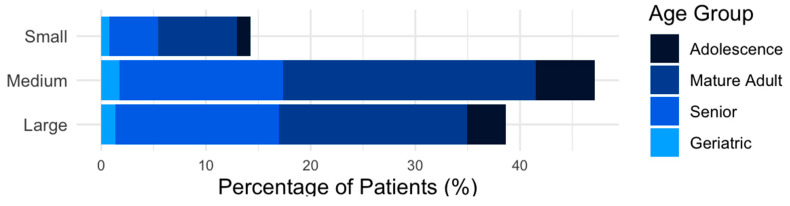
Distribution of patients by weight and age groups.

The median (range) age and weight of the dogs were 72.5 (2–199) months and 19.85 (3.5–76) kg, respectively. Distribution of patients by weight and age groups is shown in Figure 1.

**Figure 2:**
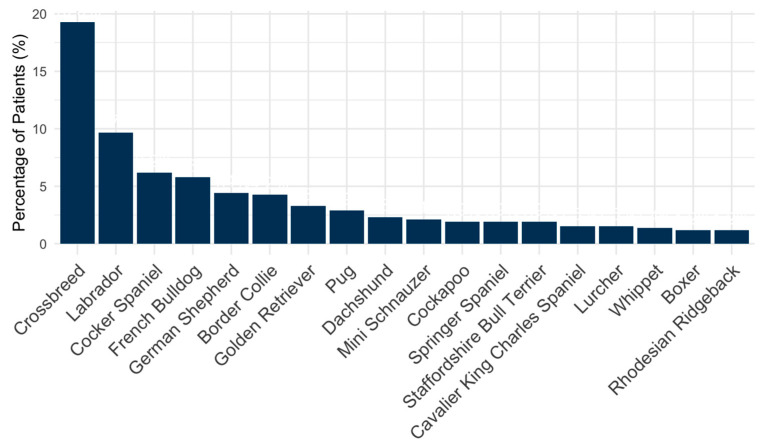
Bar chart representing the distribution of patients by breed (only the breeds accounting for ≥1% of the study population are represented).

The sample comprised 89 different breeds. Crossbreed was the most frequent breed (n = 94; 19.3%), followed by Labrador Retriever (n = 46; 9.6%), Cocker Spaniel (n = 30; 6.2%), French Bulldog (n = 30; 5.8%) and German Shepherd dog (n = 22; 4.4%). Chondrodystrophic breeds comprised 26.8% of the study population (139). The distribution of breeds accounting for ≥1% of the study population is shown in Figure 2.

**Figure 3:**
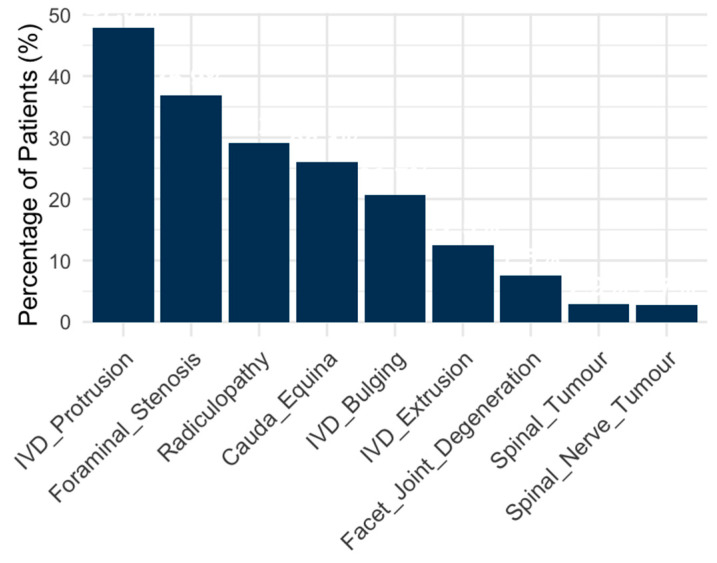
Bar chart illustrating the percentages of patients for various MRI-diagnosed pathologies (only the pathologies with >10 events in the study population are represented).

Lumbosacral pathologies were the most prevalent, affecting 66% (342) of the patients, while lumbar pathologies were identified in 36.1% (187) of the patients. The IVD protrusion was the most frequent MRI pathology (n = 248; 47.9%), followed by foraminal stenosis (n = 191; 36.9%), radiculopathy (n = 151; 29.2%), cauda equina compression (n = 135; 26.1%), and IVD bulging (n = 107; 20.7%). Notably, 52 cases of IVD bulging were not associated with either extrusion or protrusion. A distribution of common MRI pathologies is presented in Figure 3. Less frequent MRI pathologies reported were vertebral subluxation (n = 8; 1.5%), discospondylitis (as main differential diagnosis) (n = 7; 1.4%), syringomyelia (n = 4; 0.8%), sacroiliac degeneration (n = 3; 0.6%), subarachnoid diverticulum (n = 3; 0.6%), tethered spinal cord (n = 3; 0.6%), epidural empyema (n = 3; 0.6%), fused vertebrae (n = 1; 0.2%), epidural haemorrhage (n = 1; 0.2%), myelomeningocele (n = 1; 0.2%), osseous tumour (n = 1; 0.2%), lumbar epaxial myositis (n = 1; 0.2%).

Additionally, the distribution of the patients according to the number of concurrent spinal pathologies was evaluated. Among the total population, 109 patients (21%) had no pathologies, 121 patients (23.4%) presented with a single pathology, 95 patients (18.3%) had two concurrent pathologies, 101 patients (19.5%) had three pathologies, and 92 patients (17.8%) exhibited more than three concurrent pathologies. A hip pathology was concurrently present in 1.3% (7) of patients with a lumbar pathology and 4.6% (24) of those with a lumbosacral pathology.

### 3.3. Agreement Between Referring and Referral Veterinary Surgeons on Overall Pain Status

A total of 366 patients (out of 518) were considered to be in pain in this study. Cohen’s kappa coefficient indicated a moderate level of agreement between the referring and referral veterinary surgeons regarding the overall pain status (κ = 0.70; 95% CI: 0.63–0.76). The referring veterinary surgeon identified pain in 33 cases where the referral surgeon did not, and the referral surgeon identified pain in 39 cases where the referring surgeon did not.

Pain was most frequently observed during lumbosacral palpation, affecting 239 patients (46.1%), followed by lumbar palpation in 123 patients (23.7%), and hip extension in 101 patients (19.5%). Tail dorsiflexion was the least common pain response, observed in 30 patients (5.8%). In the subset of patients presenting with concurrent hip and lumbosacral pathology (n = 24; 4.6%), 14 patients (2.7%) had pain during lumbosacral palpation.

In the subgroup of the patients who underwent MRI without lumbar/lumbosacral pain (152 out of 518), coexisting clinical concerns included pelvic limb lameness (64, 42.1%), pelvic limb weakness (20, 13.1%), pelvic limb ataxia (12, 7.9%), generalised ataxia (10, 6.6%), urinary and faecal incontinence (10, 6.6%), stiffness (7, 4.6%), abnormal gait (5, 3.3%), behavioural changes (5, 3.3%), reluctance to exercise (4, 2.6%), vocalisation episodes (4, 2.6%), tremors (4, 2.6%), ambulatory paraparesis (3, 2%), focal seizures (2, 1.3%), gluteal muscle atrophy (1, 0.7%), and stiffness (1, 0.7%). Associated final diagnoses, in addition to those previously reported, included open diagnosis/unknown origin (109, 71.7%), cruciate ligament disease (4, 2.6%), elbow osteoarthritis (4, 2.6%), non-painful thoracic IVD disease (2, 1.3%), idiopathic epilepsy (2, 1.3%), road traffic accidents (2, 1.3%), BOAS-related exercise intolerance (1, 0.7%), tibial fracture (1, 0.7%), mitochondrial storage disease (1, 0.7%), polyneuropathy (1, 0.7%), meningoencephalitis of unknown origin (1, 0.7%), inflammatory bowel disease (1, 0.7%), degenerative myelopathy (1, 0.7%), hypothyroidism (1, 0.7%), lipoma interfering with articular range of motion (1, 0.7%), immune mediated polyarthritis (1, 0.7%), gluteal neuropathy (1, 0.7%), and prostatic hyperplasia (1, 0.7%).

### 3.4. Association Between Overall MRI Pathology and Overall Pain Status

**Figure 4:**
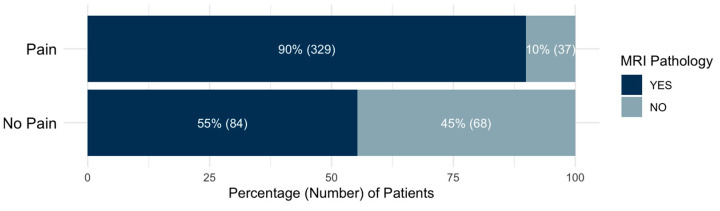
Bar chart illustrating the percentage of patients with and without MRI pathologies among those with pain and without pain.

A Pearson’s Chi-squared test of independence, with Yates’ continuity correction, was performed to evaluate the association between the overall MRI pathology and overall pain status. The results revealed a significant association between MRI pathology and pain (X^2^ = 64.684, df = 1, *p* < 0.001). These findings indicate that the patients with an MRI pathology are significantly more likely to experience pain compared to those without an MRI pathology. The effect size, Cramér’s V = 0.392, suggests a moderate association between an MRI pathology and pain, indicating that while MRI findings are linked to pain presence, additional factors likely contribute to pain perception. Figure 4 represents the distribution of MRI pathology among the patients with and without pain.

### 3.5. Age and Weight on Overall MRI Pathology and Overall Pain Status

Patients in the medium and large weight groups demonstrated a trend towards higher odds of an MRI pathology compared to the small weight group. Patients in the medium weight group showed marginally higher odds of an MRI pathology (OR = 1.81, 95% CI = [1.00, 3.26], *p* = 0.050), while the patients in the large weight group exhibited significantly greater odds (OR = 2.52, 95% CI = [1.34, 4.76], *p* = 0.004).

A similar trend was observed across the age groups, with older patients generally exhibiting higher odds of an MRI pathology compared to adolescents. Mature adults showed significantly increased odds of an MRI pathology (OR = 2.20, 95% CI = [1.17, 4.14], *p* = 0.014). Senior patients had even higher odds of an MRI pathology (OR = 2.95, 95% CI = [1.53, 5.70], *p* = 0.002), and geriatric patients displayed the highest odds (OR = 11.81, 95% CI = [1.49, 93.67], *p* = 0.021).

**Figure 5:**
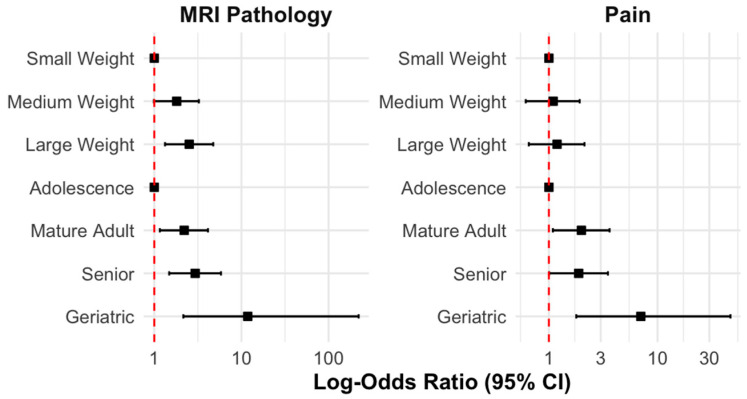
Forest plot displaying log-odds ratios (95% confidence intervals) for MRI pathology (left panel) and overall pain (right panel) in relation to weight groups (small, medium, and large) and age groups (adolescence, mature adult, senior, and geriatric).

For the overall pain, no significant association was observed between the weight groups and pain (*p* > 0.05). Regarding age, a significant association was found for older age groups. Mature adults exhibited significantly higher odds of experiencing pain compared to adolescents (OR = 1.99, 95% CI = [1.09, 3.62], *p* = 0.024), as did senior patients (OR = 1.88, 95% CI = [1.01, 3.50], *p* = 0.046). Geriatric patients demonstrated markedly higher odds of experiencing pain (OR = 7.02, 95% CI = [1.51, 32.63], *p* = 0.014). Figure 5 illustrates the relationship between weight and age groups with MRI pathology and overall pain.

### 3.6. Specific MRI Pathologies and Pain Status

Given the significant impact of age on the presence of both pathology and pain, and the significant association between weight and pathology, as demonstrated in the previously developed models, we applied restricted cubic splines (RCS) in the general, lumbosacral, and lumbar models to better capture potential non-linear relationships between age and weight, and pathology and pain. By incorporating RCS, these models more flexibly account for non-linear effects of age and weight, improving their ability to uncover complex patterns and enhance predictive performance while maintaining clinical relevance.

**Figure 6:**
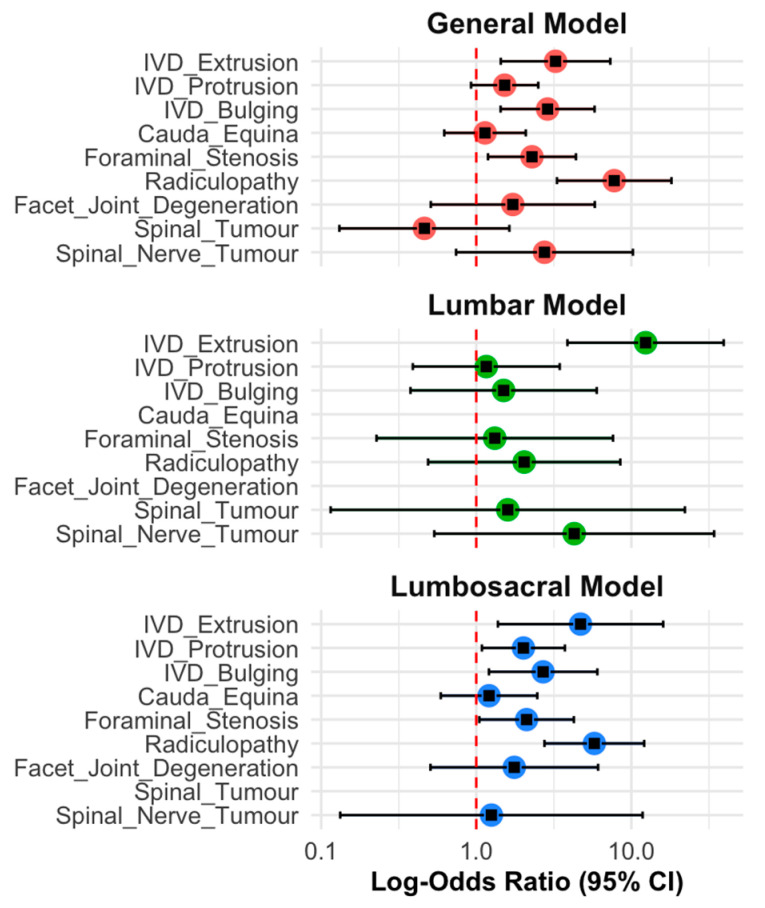
Forest plots showing log-odds ratios (95% confidence intervals) for MRI-diagnosed pathologies associated with pain across three models. The general model evaluates the associations with overall pain, the lumbar model examines lumbar pathologies and their association with lumbar pain (excluding lumbosacral pathology), and the lumbosacral model evaluates lumbosacral pathologies and their association with lumbosacral pain (excluding lumbar pathology).

In the general model (Figure 6), focused on associations between different MRI pathologies and overall pain, significant increased odds of pain were observed for IVD extrusion (OR = 3.24, 95% CI = [1.59, 6.59], *p* = 0.003), IVD bulging (OR = 2.88, 95% CI = [1.46, 5.71], *p* = 0.002), and foraminal stenosis (OR = 2.29, 95% CI = [1.19, 4.40], *p* = 0.012). Radiculopathy exhibited the strongest association among the evaluated pathologies, with patients affected by this condition having over seven times the odds of overall pain compared to those without radiculopathy (OR = 7.75, 95% CI = [3.49, 17.24], *p* < 0.001). Other pathologies, including intervertebral disc protrusion (OR = 1.52, 95% CI = [0.90, 2.57], *p* = 0.113), cauda equina compression (OR = 1.14, 95% CI = [0.64, 2.02], *p* = 0.660), facet joint degeneration (OR = 1.72, 95% CI = [0.53, 5.59], *p* = 0.382), and spinal nerve tumours (OR = 2.75, 95% CI = [0.83, 9.14], *p* = 0.130) showed positive but non-significant associations with overall pain. Spinal tumours demonstrated a negative association with overall pain (OR = 0.46, 95% CI = [0.21, 1.03], *p* = 0.064) but did not reach statistical significance.

The second model (Figure 6), focused on the associations between pathologies in the lumbar region and lumbar pain while excluding patients with a lumbosacral pathology, showed that an intervertebral disc extrusion emerged as the only significant predictor of lumbar pain (OR = 12.34, 95% CI = [4.39, 34.67], *p* < 0.001). Other pathologies, including intervertebral disc protrusion (OR = 1.16, 95% CI = [0.52, 2.54], *p* = 0.79), intervertebral disc bulging (OR = 1.50, 95% CI = [0.55, 4.10], *p* = 0.57), foraminal stenosis (OR = 1.32, 95% CI = [0.32, 5.51], *p* = 0.76), and radiculopathy (OR = 2.04, 95% CI = [0.61, 7.03], *p* = 0.33), showed positive but non-significant associations with lumbar pain. Spinal tumours (OR = 1.60, 95% CI = [0.26, 9.84], *p* = 0.73) and spinal nerve tumours (OR = 4.28, 95% CI = [0.54, 31.94], *p* = 0.17) similarly failed to achieve statistical significance. Facet joint degeneration and cauda equina compression were not included in this analysis due to their absence in the sub-cohort.

The third model (Figure 6), which assessed the associations between the pathologies localised to the lumbosacral region and lumbosacral pain while excluding patients with lumbar pathology, identified significant predictors including IVD extrusion (OR = 4.69, 95% CI = [1.80, 7.28], *p* = 0.013), IVD protrusion (OR = 2.01, 95% CI = [1.14, 3.56], *p* = 0.026), IVD bulging (OR = 2.62, 95% CI = [1.22, 5.62], *p* = 0.016), foraminal stenosis (OR = 2.11, 95% CI = [1.06, 4.22], *p* = 0.037), and radiculopathy (OR = 6.12, 95% CI = [2.88, 11.74], *p* < 0.001). Cauda equina compression (OR = 1.17, 95% CI = [0.63, 2.19], *p* = 0.604), facet joint degeneration (OR = 1.90, 95% CI = [0.62, 5.83], *p* = 0.375), and spinal nerve tumours (OR = 1.66, 95% CI = [0.50, 5.46], *p* = 0.844) did not show significant associations with lumbosacral pain. Spinal tumours were not included in this analysis due to their absence in the sub-cohort.

### 3.7. Clinical Examination Findings and Pain and Pathology

Two binary logistic regression models incorporating RCS for both age and weight were developed to capture the potential non-linear relationships with pain and pathology, exploring the associations between specific clinical pain examination findings and pain or pathology outcomes.

In the first model, the relationship between the pain during hip extension and the combined presence of lumbosacral pain and pathology was examined, excluding cases of hip pathology. Pain during hip extension was identified as a significant predictor, increasing the odds of the combined presence of lumbosacral pain and pathology (OR = 1.98, 95% CI = [1.14, 3.45], *p* = 0.009). In the second model, the association between pain during lumbosacral palpation and the combined presence of hip extension pain and a hip pathology was assessed after excluding cases of lumbosacral pathology. This analysis revealed a significant association, with the dogs exhibiting both hip pathology and pain being more likely to present with lumbosacral pain on palpation (OR = 1.44, 95% CI = [1.03, 3.98], *p* = 0.034).

### 3.8. Model’s Performance

Results for the Hosmer–Lemeshow test, AIC, Deviance, and C-statistics for all models are summarised in Table 1.

The models assessing weight and age on MRI pathology and pain demonstrated adequate calibration and poor discriminatory power. Weight and age on MRI pathology had better fit-to-complexity balance, as reflected by its lower AIC.

**Figure 7:**
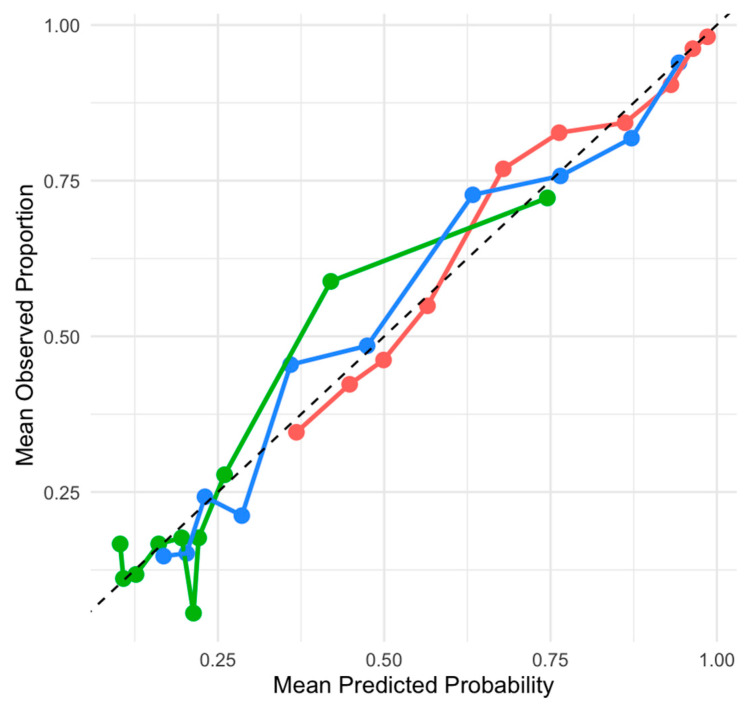
Calibration plots showing the relationship between mean predicted probabilities and mean observed proportions across deciles for the general model, lumbar model, and lumbosacral model. The dashed diagonal line represents perfect calibration, where the predicted probabilities equal observed proportions.

The general model evaluating the MRI-diagnosed pathologies and overall pain demonstrated excellent discriminatory power and adequate calibration. The lumbosacral model displayed a similarly excellent discriminatory ability and adequate calibration. Its fit-to-complexity trade-off was superior to that of the general model. The lumbar model showed acceptable discriminatory power and adequate calibration. It had the lowest AIC, indicating the best fit-to-complexity balance. However, its discriminatory power was weaker than the lumbosacral and general models. The calibration performance of the general, lumbar, and lumbosacral models is visually represented in Figure 7. The calibration plot compares the mean predicted probabilities with the mean observed proportions across the deciles for each model. While all three models exhibit adequate calibration, the general and lumbosacral models demonstrate superior calibration across most probability ranges, closely following the ideal calibration line (dashed diagonal). The lumbar model, though still within an acceptable range, shows a tendency to overestimate the probabilities at higher predicted values.

Finally, the clinical pain examination models demonstrated adequate calibration and poor discriminatory power. The lumbosacral palpation model had better fit-to-complexity balance, as reflected by its lower AIC.

## 4. Discussion

A multivariable analysis is essential in developing predictive models that estimate the probability of clinical outcomes based on multiple interacting factors. These models aid clinicians in identifying meaningful associations, reducing reliance on subjective clinical judgement, and supporting evidence-based decision-making [16,17].

A multivariable approach was essential in our study, given the high prevalence of concurrent spinal pathologies in the study population. More than a third of patients (37.3%) presented with three or more concurrent spinal abnormalities, highlighting the complexity of assessing caudal spinal pain. To account for potential confounding and interaction effects, we employed multivariable logistic regression models, allowing for the simultaneous adjustment of multiple factors and providing a more precise estimation of the independent association of each pathology with pain. Given the significant influence of age on both spinal pathology and pain, as well as the significant association between weight and spinal pathology, RCS were incorporated for both variables to capture potential non-linear relationships with these outcomes. This approach improved model flexibility while maintaining interpretability, ensuring that age- and weight-related effects were appropriately modelled. While weight was not significantly associated with pain, its inclusion in the models allowed for a more comprehensive assessment of its potential influence on pathology-related pain. Given the higher prevalence of lumbosacral pathologies in our sample, we developed separate lumbar and lumbosacral models to build on the findings from our general model. By isolating regional effects, this approach provided a more focused analysis of MRI-diagnosed pathologies in each anatomical area, helping to clarify how individual pathologies contribute to pain.

The multivariable logistic regression models demonstrated varying levels of performance across different model groups. The models assessing weight and age on MRI pathology and pain exhibited poor discriminatory power but adequate calibration. Within this model group, the MRI pathology model demonstrated a better fit-to-complexity balance, as indicated by its lower AIC. Despite their limitations in classification accuracy, these models provide insight into the demographic factors influencing MRI-detected pathology and pain presence. The general model, which assessed MRI-diagnosed pathologies and overall pain, exhibited excellent discriminatory power and adequate calibration. The lumbosacral model, focusing on pathologies within the lumbosacral region, performed similarly, with excellent discriminatory ability and a superior fit-to-complexity trade-off, reinforcing its utility as a robust predictive model. The lumbar model demonstrated acceptable discriminatory power and adequate calibration, with the best fit-to-complexity balance among all models, as indicated by its lowest AIC. However, its lower discriminatory power suggests a more limited ability to classify pain status despite its optimised model fit. The clinical pain examination models, assessing pain during a hip extension and a lumbosacral palpation, exhibited poor discriminatory power but adequate calibration. Within this model group, the hip extension model demonstrated a better fit-to-complexity balance, as reflected by its lower AIC. These findings underscore the limited predictive utility of clinical pain examination alone in differentiating between hip and lumbosacral pain, highlighting the need for integrated diagnostic approaches combining clinical examination with imaging and additional diagnostic modalities. All interpretations of model performance were conducted in accordance with the established statistical literature [16,17], ensuring compliance with the recommended methodologies for evaluating discrimination, calibration, and model fit.

Radiculopathy emerged as the strongest predictor of pain in both the general and lumbosacral models, highlighting its role as a primary pain generator. IVD extrusion, foraminal stenosis, and IVD bulging also showed significant associations with pain in these models. Conversely, IVD protrusion reached significance only in the lumbosacral model, suggesting that its clinical impact may be more pronounced in that region. Other pathologies, such as cauda equina compression, facet joint degeneration, and spinal nerve tumours, did not achieve statistical significance, highlighting the complexity of interpreting MRI findings and underscoring that these pathologies may contribute to pain only in certain contexts.

In the lumbar model, IVD extrusion emerged as the only significant predictor of lumbar pain, highlighting its substantial role in patients with exclusively lumbar pathology. Other potential contributors such as intervertebral disc protrusion, foraminal stenosis, bulging and radiculopathy showed positive but not significant associations with lumbar pain. This underscores the importance of disc extrusion in generating pain in the lumbar spine, where the spinal canal primarily contains the spinal cord.

The anatomical differences between the lumbar and lumbosacral regions are likely key to understanding these findings. In the lumbar spine, the spinal cord occupies most of the spinal canal and extends to approximately the L6-L7 vertebra in dogs [18,19]. This region has limited reserve space and sparse epidural fat, leaving the spinal cord particularly vulnerable to compression or injury from herniated intervertebral discs. In contrast, the lumbosacral region contains the cauda equina rather than the spinal cord, and its comparatively large epidural fat content provides a buffering effect against compression. As a result, a more substantial disc herniation is typically required to cause significant cauda equina compression [20]. This buffering capacity may explain the absence of a significant association between cauda equina compression and pain in our study.

The lumbosacral joint, being the most mobile segment of the lumbar spine, plays a critical role in force transfer between the spine and pelvis. This high degree of mobility subjects the joint to repetitive strain and mechanical stress, predisposing it to instability and degeneration. These degenerative changes affect not only the intervertebral disc but also extend to peripheral structures such as the facet joints and ligaments, contributing to the array of pathologies encompassed within the broad diagnosis of degenerative lumbosacral stenosis (DLSS) [8,10]. These structural changes have significant implications for the neural elements in this region. While the cauda equina benefits from the buffering provided by the epidural fat within the lumbosacral canal, the intervertebral foramina lack such a protective reserve. Consequently, spinal nerves exiting through the foramina are particularly vulnerable to compression and dynamic impingement, especially in the presence of foraminal stenosis. This interplay of joint mobility, degenerative changes, and anatomical constraints underscores the lumbosacral region’s predisposition to radiculopathy and likely explains its strong association with pain observed in our study.

Degenerative changes leading to IVD protrusion are typically localised within the dorsal or dorsolateral annulus fibrosus [11] and can result in herniation of the IVD towards the spinal canal and intervertebral foramina. IVD protrusions have previously been associated with pain in dogs, and although they are far less common in the caudal lumbar region compared to the thoracolumbar region, when present, this pain is often linked to radiculopathies [21,22].

In this study, IVD bulging was reported alongside IVD extrusion or protrusion in most cases and was used as a non-specific term to describe any form of IVD disease involving the displacement of the intervertebral disc beyond its normal boundaries. The exclusive use of “IVD bulging” as a diagnosis may reflect instances where imaging findings lacked definitive features to distinguish between extrusion and protrusion, or cases where subtle disc deformations were observed without clear evidence of herniation into the spinal canal or foramina. While criteria have been proposed to aid in differentiating the types of IVD herniation in large-breed dogs with thoracolumbar IVD herniation [23], no guidelines currently exist for characterising IVD herniation in the lumbar and lumbosacral regions of dogs. This lack of standardised criteria underscores the need for clear guidelines to improve the accurate characterisation of intervertebral disc disease in the lumbosacral region, particularly in cases with overlapping or ambiguous findings.

In human medicine, although facet joint degeneration has been reported in up to 60% of young adults [24], studies suggest that it can remain asymptomatic in up to 18% of young adults and as many as 80% of elderly patients [7]. However, facet joint degeneration has been identified as the source of pain in up to 44% of patients presenting with LBP [25,26,27,28,29].

In veterinary medicine, a study of military German Shepherd dogs undergoing MRI revealed a 37.9% prevalence of facet joint degeneration [30]. Dogs with lumbosacral pain were more likely to have facet joint degeneration affecting three or more vertebral locations, suggesting a potential role of facet joint degeneration as pain generator. However, the authors employed a univariable statistical approach in a population with multiple concurrent spinal pathologies. Consequently, the authors recommended further research to better elucidate the specific contribution of facet joint degeneration to pain in similar cases. In our study, facet joint degeneration did not emerge as a statistically significant predictor of pain in either the general cohort or the lumbosacral-focused analyses. This finding aligns with previous human and veterinary reports indicating that a substantial proportion of facet joint changes can remain asymptomatic.

Tumours affecting the spinal cord or spinal nerves can result in a wide variety of clinical signs, including pain, which may or may not be evident on palpation or behavioural assessment. Tumour-associated pain can resemble that caused by other spinal conditions, such as intervertebral disc herniation and radiculopathies, as tumours can exert both mechanical compression and chemical irritation on the spinal cord and nerves. However, the clinical presentation varies depending on several factors, including tumour location (extradural, intradural, or intramedullary), growth characteristics, biological behaviour, and whether the tumour is situated cranially or caudally, or associated with the cervical or lumbar intumescence. Additionally, the presence of concurrent spinal or musculoskeletal conditions may further complicate the attribution of pain specifically to the tumour [31,32].

In our study, 15 dogs were diagnosed with spinal tumours and 14 with spinal nerve tumours. Although these counts surpassed our minimum threshold of ten events per variable for inclusion in the regression models, the limited event numbers reduced statistical power and may have widened the confidence intervals. This limitation could have also affected our ability to detect a true association between these pathologies and pain. The resulting wide confidence intervals for the odds of pain with spinal and spinal nerve tumours may also reflect substantial heterogeneity in tumour characteristics, location, progression status and clinical presentation. In the general model, spinal tumours were associated with a negative, but not statistically significant relationship with pain, whereas spinal nerve tumours showed a positive, yet similarly non-significant association. These variable trends may partly explain the lack of definitive findings. Larger, more focused studies, ideally stratifying tumour types, anatomical regions and disease stages, are needed to better understand the role of these tumours as pain generators.

The prevalence of sacroiliac pathology in large breed dogs has been reported to range from about 50% to 100% in young dogs and can reach nearly 100% in older dogs [13,33]. However, because these studies provided limited information on the clinical context and pain status, no evaluation of potential associations between sacroiliac disease and pain was established. Both reports also noted the frequent presence of concurrent pathology. In one study, 74% of dogs had coexisting lumbosacral or pelvic findings, leading the authors to suggest that this overlap could complicate the efforts to determine the specific contribution of sacroiliac pathology to lumbosacral pain [13]. Sacroiliac degeneration was rarely reported in our study. While this difference may partly reflect the broader range of breeds included, the considerable discrepancy could also be attributed to radiologists prioritising the reporting of lesions they deemed clinically relevant.

In this study, spondylosis deformans was not systematically recorded because the authors did not initially regard it as a primary contributor to pain. Consequently, its potential influence on lumbar and lumbosacral pain may have been underestimated. Spondylosis deformans frequently affects multiple regions of the canine spine, with reported prevalence ranging from about 5% to 85% in older individuals of susceptible breeds [34,35,36,37,38].

Enthesophytes typically develop along the ventral and lateral aspects of the vertebrae rather than dorsally, which explains why spinal cord compression is rarely observed. However, some authors propose that extensive new bone formation can lead to nerve impingement and radicular pain, particularly when enthesophytes extend toward the dorsolateral aspects of the vertebrae [37,38].

Many dogs with spondylosis deformans remain asymptomatic, and the frequent coexistence of other spinal disorders further complicates the direct attribution of pain to this condition [20,35,36,38,39]. Future studies should consider systematically recording spondylosis deformans to clarify its clinical significance and distinguish its effects from those of concurrent spinal conditions. If spondylosis-mediated lumbar or lumbosacral pain were primarily due to radiculopathy, it would likely have been captured by the radiculopathy variable in our analysis.

Beyond the documented forms of IVD herniation in our study, we did not record IVD degeneration in the discs that otherwise appeared structurally normal, where no herniation or displacement could compress neural or non-neural tissues. This omission may have led to an underestimation of a potential association between IVD degeneration and discogenic pain in patients presenting with pain but no identifiable MRI pathology. Although the precise mechanisms underlying discogenic pain remain incompletely understood, current evidence points to a complex interplay of inflammatory, mechanical, and neurogenic factors. In patients with IVD degeneration, inflammatory processes arising from the deteriorating disc can promote aberrant innervation of the annulus fibrosus and nucleus pulposus, triggering nociceptive signalling. Structural changes, such as fissures in the annulus, reduced disc height, and an overall loss of disc integrity, can then intensify mechanical stress on these newly formed nerve endings. Over time, continuous stimulation of peripheral nociceptors may lead to peripheral sensitisation, in which the nerve fibres become increasingly reactive to both painful and non-painful stimuli. Repeated activation can ultimately result in central sensitisation within the dorsal horn of the spinal cord, further amplifying pain perception. In combination, these processes appear to foster a cycle in which inflammation, aberrant nerve ingrowth, compromised disc architecture, and heightened sensitisation all contribute to discogenic pain [40,41]. In veterinary medicine, discogenic pain remains largely unexplored, and the lack of targeted research on this topic in dogs hinders our understanding of its pathophysiological mechanisms, diagnostics, and clinical significance. Anatomical differences, such as the reduced innervation of canine IVD compared to humans, may limit the applicability of mechanisms described in human medicine [42]. Furthermore, the absence of direct patient feedback, which forces reliance on behavioural observations and clinical examination, complicates the study of discogenic pain in dogs. Further research is therefore needed to clarify the potential implications of discogenic pain in canine patients, particularly in cases where spinal pain is present, but MRI findings do not extend beyond evidence of IVD degeneration.

The exclusion of low-prevalence pathologies such as syringomyelia, epidural empyema, and tethered spinal cord syndrome from our statistical models does not imply they lack clinical relevance. Their low frequency simply limited our ability to include them in the regression analyses. Clinicians should remain mindful that these conditions may still contribute significantly to lumbar or lumbosacral pain and may require a tailored diagnostic and therapeutic approach when suspected. Additionally, it is important to emphasise that a presumptive MRI diagnosis often requires histopathological confirmation. However, given the benefit–risk ratio, histopathological evaluations may be impractical in many patients.

Our analysis revealed that patients with an MRI pathology were significantly more likely to experience pain compared to those without MRI pathology. However, 20% (84 out of 413) of the patients with an MRI pathology were categorised as not experiencing pain. This aligns with the previous findings in both human and veterinary medicine, which indicate that not all patients with lumbar and lumbosacral spinal pathology will necessarily show signs of pain [4,5,6,7,9]. Interestingly, 10% (37 out of 366) of the patients who experienced pain included in our study exhibited spinal pain despite the absence of detectable MRI pathology. Several factors may explain this observation, including the inherent limitations of MRI in identifying all spinal pathologies, potential omissions of relevant sequences in the MRI acquisition protocol, and the possibility of non-spinal conditions mimicking spinal pain. Additionally, differences in the interpretation of imaging findings, with a focus on reporting lesions deemed most clinically relevant, could contribute to this phenomenon. Beyond a structural pathology, pain without detectable macroscopic changes may also arise from altered pain states, such as central sensitisation or nociplastic pain [1]. This aligns with the findings in human medicine, where chronic low back pain often persists despite the absence of identifiable structural abnormalities, with biopsychosocial factors playing a significant role in pain perception and persistence. These findings underscore the importance of prioritising clinical signs over imaging findings when assessing and managing spinal pain.

In human medicine, both older and larger patients have a significantly higher odds for spinal pathology and LBP. Increased body mass and obesity are recognised factors for developing spinal pathology-related LBP, primarily due to increased mechanical loading and stress on the spinal joints and intervertebral discs. Additionally, obesity promotes states that facilitate pain, and is often associated with other risk factors, such as a sedentary lifestyle, further contributing to LBP [43,44,45]. Age is associated with the deterioration of the musculoskeletal system, leading to a decline in mobility and independence. This deterioration is often accompanied by reduced physical activity, which, when maintained at moderate levels, serves as a protective factor against LBP. Additionally, age-related changes in peripheral and central nervous systems affect pain processing, facilitating nociceptive signalling to higher centres and reducing the efficiency of the descending inhibitory pain pathways [45,46,47].

In veterinary medicine, the prevalence of spinal disease is greater in dogs of both a large breed size and an advancing age [8,9,10,11,12,13]. However, the influence of age on the odds of developing lumbar or lumbosacral pain has not been extensively studied. Our analysis, consistent with the findings in human medicine, revealed that the odds of experiencing pain progressively increased from maturity to seniority when compared with adolescence, reaching statistical significance in geriatric patients.

Hip extension is commonly used as a diagnostic manoeuvre in clinical examinations, yet it has the potential to elicit pain from two main distinct sources: the hip joint itself or the lumbosacral region, where mechanical stress during hip extension may exacerbate an underlying pathology [8,14,15]. This overlap creates diagnostic uncertainty, requiring clinicians to determine whether the pain originates from hip pathology or lumbosacral disease. Additionally, the potential for lumbosacral palpation to trigger pain due to mechanical loading on the hip joints has not been widely considered. To investigate these scenarios systematically, we formulated two hypotheses. First, we examined whether pain during a hip extension in dogs without a hip pathology was associated with lumbosacral pain in those with lumbosacral disease. Second, we explored whether dogs with pain during a hip extension and a hip pathology, but no lumbosacral pathology were nonetheless prone to exhibit pain on lumbosacral palpation. Our results indicated that in dogs without a hip pathology, pain during a hip extension was associated with a higher likelihood of both lumbosacral pain and pathology. Conversely, dogs with a hip pathology and pain on hip extension, but no lumbosacral pathology, still exhibited an increased likelihood of pain elicited during lumbosacral palpation. It is important to acknowledge that characterising patients as lacking a hip pathology based on the MRI findings alone may have underestimated mild or early-stage hip pathology in our study, as MRI is not the gold standard for assessing the coxofemoral joints.

Clinicians should view these findings as a reminder that pain during a hip extension is not exclusively diagnostic of a significant hip pathology or a lumbosacral pathology on its own. In dogs without a hip pathology, pain elicited during a hip extension should raise suspicion of a significant lumbosacral pathology, warranting further neurological assessment and imaging. Conversely, in dogs with a significant hip pathology, especially when the MRI findings do not indicate lumbosacral abnormalities, clinicians should remain alert to the possibility of referred or secondary lumbosacral pain. A careful, integrated approach, combining orthopaedic and neurological examinations, imaging when indicated, and a close observation of the patient’s response to therapeutic interventions ensures that treatment is appropriately targeted to the actual source/s of pain.

In our analysis, lumbosacral pain was assessed exclusively using the variable “pain during lumbosacral palpation” to prevent the pain on hip extension from acting as a confounding factor. This approach minimised the risk of attributing spinal pain to patients with or without a spinal pathology when the actual pain source was a hip pathology. However, our findings suggest that referred or secondary lumbosacral pain may occur in dogs with a significant hip pathology, posing a risk of misattributing the pain elicited during a lumbosacral palpation to a spinal pathology. Nevertheless, given that only 4.6% of the study population had concurrent hip and lumbosacral pathology, and that only a subset of these cases exhibited lumbosacral pain, the potential confounding effect is expected to be minimal. Consequently, its impact on the robustness of the multivariable models is unlikely to be significant.

Recognising which MRI findings have the strongest associations with pain is crucial for clinical decision-making, helping veterinarians determine the most appropriate site for treatment, whether through pharmacological or non-pharmacological therapies, interventional pain management, or surgical interventions.

This is particularly relevant in the surgical treatment of the IVD disease, where the type of disc pathology influences surgical planning. IVD extrusions are typically managed with hemilaminectomy, a relatively straightforward decompressive procedure, whereas IVD protrusions often require more complex surgical techniques, such as lateral corpectomy and vertebral stabilisation, and dogs with IVD protrusions are at an increased risk of early postoperative neurological deterioration compared to dogs with extrusions [23]. Similarly, in cases of lumbosacral pain, identifying whether radiculopathy, foraminal stenosis, IVD extrusion, or IVD protrusion is the main pathology associated with the clinical presentation allows for more precise therapeutic targeting.

Interventional pain management serves as a valuable diagnostic and therapeutic tool in this context. While interventional pain management is commonly considered within the spectrum of conservative treatments due to its minimally invasive nature, it can also serve as an intermediate step between conservative and surgical approaches. By providing targeted pain relief, these interventions may help delay or even avoid the need for surgery in selected cases. When pain cannot be clearly attributed to a single source (particularly in cases where hip osteoarthritis and a lumbosacral pathology overlap), techniques such as selective nerve blocks and intra-articular injections help localise the primary pain generator [48,49]. When used for diagnostic purposes, the goal is to achieve temporary pain relief, confirming whether a specific anatomical structure, such as a nerve root or joint, is responsible for the patient’s clinical signs. This is particularly relevant in preventing unnecessary interventions based solely on imaging findings, given that, in addition to the challenge of identifying the clinical relevance of concurrent pathologies, structural abnormalities do not always correlate with clinical pain.

As a therapeutic modality, image-guided caudal and transforaminal epidural injections are widely used in human medicine for managing lumbosacral disc herniation and radicular pain [50,51] and similar techniques have been applied in veterinary medicine [52,53,54,55]. However, significant variability exists in veterinary studies regarding patient selection, procedural techniques, drugs administered, peri-procedural management, and reported outcomes. This heterogeneity makes it challenging to draw definitive conclusions regarding their long-term efficacy in dogs.

Current recommendations for managing canine degenerative lumbosacral stenosis (CDLSS) prioritise conservative approaches, with surgical interventions reserved for cases refractory to conservative management [10,15]. Surgical techniques aim to relieve neural compression and stabilise the lumbosacral joint, although outcomes vary depending on the severity of pathology and technique employed [10,14,15]. As with conservative interventions, variability in study methodologies, including patient selection, techniques, and outcome reporting, further complicates the ability of veterinarians to draw definitive conclusions regarding the optimal management strategies for canine lumbar and lumbosacral spinal pain.

Pain categorisation in this study was based exclusively on subjective non-standardised clinical examination findings, excluding behavioural manifestations reported by the owners. This exclusion may have resulted in an underestimation of the painful population, potentially reducing the sensitivity of the study to identify the associations between certain pathologies and intermittent pain, or pain not being detected during the clinical examination. While this approach may have underrepresented the true prevalence of pain within the study population, it likely reduced the incidence of false positives, thereby improving the specificity of pain classification. This more stringent categorisation may have enhanced the ability of the statistical models to identify pathologies truly associated with pain.

Pain assessment was performed independently by two groups: the referring veterinary surgeons and the referral veterinary specialists, with moderate agreement observed between their evaluations. Differences between the observations could have been attributed not only to variability in the performance and interpretation of their examinations but also to the fluctuations in the patients’ pain status at the time of each assessment and the prescription of analgesic medications prior to referral. Furthermore, because there are no established guidelines for a standardised clinical objective grading system in dogs with lumbar/lumbosacral pain, the attending veterinarians relied on their clinical judgement. Evaluating the specific assessment methods was beyond the scope of this study, so variability in how the pain was elicited and interpreted remains possible. Patients were classified as experiencing pain if either of the assessors identified them as such, which likely mitigated the impact of variations in the agreement between observers and ensured a more inclusive identification of painful cases.

The absence of a purpose-built control group comprising dogs without pain, where MRI was performed despite not being clinically indicated, represents a limitation of this study. However, subjecting dogs to general anaesthesia solely for the purpose of creating a control group is not ethically justifiable due to the associated anaesthetic risks. This differs from human medicine, where MRI can often be conducted without the need for general anaesthesia. The population included in this study represents a clinically relevant cohort, encompassing a broad range of ages, weights, and breeds (including both chondrodystrophic and non-chondrodystrophic breeds) and comprising dogs with and without pain, where MRI was clinically indicated.

MRI is widely regarded as the gold standard for assessing spinal pathologies [2]. However, conventional T1- and T2-weighted sequences have limitations in evaluating small and specific anatomical regions. Slice positioning cannot be predetermined with millimetric precision, potentially leaving “gaps” of tissue between slices that may exclude critical structures. This limitation is particularly relevant in small companion animals, where the small size of anatomical structures at the neurovascular bundle magnifies the impact of spatial errors [56]. These challenges may be even more relevant when compared to humans, whose larger anatomical structures reduce the relative margin of spatial error. Coronal MRI using a three-dimensional fast-field echo with a water-selective excitation technique is considered superior to dorsal planar short tau inversion recovery (STIR) imaging for assessing the canine foraminal and extraforaminal regions of the spinal nerves. This superiority is attributed to its enhanced spatial resolution, improved contrast, and fat suppression capabilities, which allow for precise visualisation of small, discrete regions, and improves the detection of neural pathologies that might otherwise go unnoticed with standard T1- and T2-weighted imaging in dogs [57,58]. As such, only the MRI studies that included 3D water excitation sequences were considered in this study. While this approach improved the ability to identify clinically relevant neural pathologies, it may have introduced a selection bias towards patients with radiculopathies, as this imaging technique is frequently employed in cases with suspected nerve involvement. To address this potential bias, multivariable logistic regression models were employed, accounting for the influence of multiple variables simultaneously. This statistical approach ensured that the pathologies identified as significant were those most strongly associated with pain in the study population, thereby enhancing the robustness and clinical relevance of the findings.

A key limitation of our study is that we did not re-review the MRI studies with a standardised framework specifically tailored to differentiate between the subtler categories of intervertebral disc herniation and other possible spinal pathologies. While a systematic re-review might have yielded a more robust dataset, the lack of universally accepted guidelines for classifying spinal pathologies in the lumbar and lumbosacral regions of dogs could have generated discrepancies between the original radiological reports and the re-assessed findings. Consequently, such an approach might not have necessarily improved the robustness or consistency in pathology classification.

## 5. Conclusions

This study provides valuable insights into the complex interplay between spinal pathologies, demographic factors, and pain in dogs with lumbar and lumbosacral MRI findings. Using a multivariable approach, we identified IVD extrusion as the primary pathology associated with lumbar pain, while radiculopathy had the strongest association with lumbosacral pain. These findings reinforce the importance of recognising which MRI pathologies are most strongly associated with pain, aiding the clinicians in making informed decisions regarding conservative, interventional, or surgical management.

Distinguishing the primary source of pain in patients with concurrent lumbar, lumbosacral, and orthopaedic pathologies remains a challenge, particularly given the overlap of pain responses during clinical examination. This diagnostic uncertainty highlights the role of interventional pain management as a complementary approach, providing a means to assess the clinical relevance of the different pathologies and refine treatment decisions. Further research focused on refining MRI criteria for diagnosing clinically relevant pathologies, advancing interventional pain management and surgical techniques, and evaluating the long-term outcomes of both is needed to improve our understanding and management of lumbar and lumbosacral pain in dogs.

## Figures and Tables

**Table 1:** Model assessment results, including number of subjects, deviance, AIC, C-statistics, and Hosmer–Lemeshow test outcomes for all models.

Model	Subjects (n)	Null Deviance	Residual Deviance	AIC	C-Statistic	Hosmer-Lemeshow (χ^2^, *p*-Value)
Weight and Age on MRI Pathology	518	522.27	500.72	512.72	0.621	χ^2^ = 9.57, *p* = 0.088
Weight and Age on Pain	518	626.99	616.96	628.96	0.564	χ^2^ = 0.208, *p* = 0.995
General Model	518	626.99	493.82	529.81	0.808	χ^2^ = 6.57, *p* = 0.584
Lumbar Model	176	200.11	169.84	193.84	0.732	χ^2^ = 9.275, *p* = 0.732
Lumbosacral Model	331	458.79	345.76	379.76	0.819	χ^2^ = 3.658, *p* = 0.887
Clinical Pain (Hip Extension and LS Pain/Pathology)	474	638.34	598.93	618.93	0.664	χ^2^ = 12.722, *p* = 0.121
Clinical Pain (LS Palpation and Hip Pain/Pathology)	175	169.45	158.91	178.91	0.634	χ^2^ = 12.912, *p* = 0.1149

## Data Availability

The data that support the findings of this study are available from the main author upon reasonable request.
